# Exploration of Extension Research to Promote Genetic Improvement in Cattle Production: Systematic Review

**DOI:** 10.3390/ani14020231

**Published:** 2024-01-11

**Authors:** Patricia Menchon, Jaime K. Manning, Dave L. Swain, Amy Cosby

**Affiliations:** 1 Institute for Future Farming Systems, School of Health, Medical and Applied Sciences, CQUniversity Australia, Rockhampton, QLD 4701, Australia; j.k.manning@cqu.edu.au (J.K.M.); dave.swain@terracipher.com (D.L.S.); a.cosby@cqu.edu.au (A.C.); 2 TerraCipher, 337 Laurel Bank Rd., Alton Downs, QLD 4702, Australia

**Keywords:** adoption, genetic, cattle, technology acceptance

## Abstract

**Simple Summary:**

Genetic improvement in the cattle production industry is a driver of productive, economic and sustainable improvements. Farmers have genetic tools that make it easier for them to make decisions considering their social and productive reality. Although there are new techniques and tools to improve livestock genetics, and despite efforts to promote their use through agricultural extension, there is still a gap between the creation of these tools and their actual use by livestock farmers. This review was conducted to understand how genetic improvement tools in livestock production are being promoted globally. Most studies carried out surveys or interviews with farmers and stakeholders on both social and productive topics. Although social factors are known to affect whether these improvements are adopted or not, the use of social theories to understand this is still in its incipient. More research is needed to understand how to successfully promote the use of these genetic tools in specific productive regions.

**Abstract:**

In the cattle industry, tools for genetic improvement play a crucial role in animal selection. The changing circumstances faced by farmers and the significant part agricultural extension plays in these changes must be considered. Despite progress in genetic selection tools and the push for their adoption through extension services, a disconnect persists between the development of new strategies and tools for genetic improvement and their adoption by livestock farmers. This systematic review is designed to globally investigate the methodology and outcomes of extension research aimed at advancing genetic improvement in beef cattle. Adhering to PRISMA guidelines, a search was conducted across four databases for studies published from January 2012 to June 2023. Twenty-one articles were selected and reviewed. The research design in the articles predominantly employed mixed methods, utilizing both quantitative and qualitative approaches. While social factors are acknowledged as influencers in the adoption process, the application of theories or frameworks from social sciences is still in its early stages. To successfully implement extension activities that promote the use of genetic tools in cattle for a specific production region, more participatory research is required where farmers are actively involved.

## 1. Introduction

Genetic improvement is a key factor in the economic sustainability of beef production. Banks [1] summarizes the decision process when embarking on genetic improvement in three steps: defining the objective (what traits are important and the direction of change desired); identifying animals with superior genetics according to the objectives; selecting those animals, mating them and producing offspring. Genetic improvement tools are used to support the selection of animals with the desired characteristics and facilitate the subsequent transmission to their offspring [2]. In the cattle industry, genetic tools can be categorized by when they are used with respect to the life of the animal. This distinction is important because it affects not only the rate of generational genetic gain for a specific trait but also the effectiveness [3]. For example, genetic evaluations that incorporate estimated genomic values (GEBV) as a selection tool for traits such as fertility and growth allow selection earlier in the life of the animal when compared with genetic evaluations that use estimating breeding value (EBV) alone [4,5,6]. Additionally, these selection tools are more effective than exclusive selection for phenotypic traits in the adult stage of the animal. Therefore, the adoption of genetic tools in cattle has an impact on the direction and speed of genetic progress.

The adoption of technology in agriculture is a broad concept that includes its development, dissemination and use by the end-user on farms. The adoption of technology is presented as a dynamic process [7,8] that demands a holistic vision [9] that pursues the general objective of increasing productivity, efficiency and profitability. The theoretical models and frameworks provide researchers with an ordered way to view and analyze the process of adoption and the factors that drive them [10]. There are a variety of models that consider, for example, acceptance of use, sustained use, intensity of use, economic risk or information management [10,11]. User acceptance is the first stage in the process of adoption, which is defined by the intention to use a technology [12]. The genetic gain achieved in cattle production systems depends on the intentions first and consequent decisions that farmers make when selecting sires and heifers as replacements. The complexity of decision making in cattle breeding is also influenced by the intrinsic factors of the farmer (e.g., behavior, expectances, preferences) and by external factors such as the quantity, diversity and format in which the genetic information of the animals is provided [13]. To address this complexity, genetic selection tools are promoted that support farmers to make decisions to improve economically important traits [14].

It is crucial to consider the context that farmers face in the processes of change [15] and agricultural extension has a key role in this process. Agricultural extension is defined as public and private sector activities that encourage human resource development, education, attitude change, technology transfer and information gathering and dissemination [16]. Extension activities that aim to encourage the use of genetic tools are based on farmers being aware not only of the tools themselves, but also of the importance of the traits to choose in the genetic selection of animals. Consideration of the productive and economic conditions, as well as the social behavior of farmers, is essential for extension services to play an important role in disseminating information and promoting technology use on farms [15,17,18,19]. The effectiveness of agricultural extension activities through the development of trust, in which farmers participate as the main source of innovation [15], is a strategic approach to improving adoption outcomes, including of genetic tools. Further research is necessary to consolidate the evidence for the efficacy of different extension approaches, especially related to the adoption of genetic tools.

Despite the technological advances in genetic selection and the encouragement to adopt through extension services, there is still a gap between the development of new strategies and tools for genetic improvement and their use by cattle farmers. In recent years, there has been increasing interest in understanding the barriers and motivations in the process of adopting new technologies amongst farmers [8,10,12,20]. Educational reforms are also proposed to address the new digital skills and use of technological tools that are required in the workforce [21]. However, some livestock industries, such as the beef sector, when compared with the dairy sector, require more support to adopt technologies such as genetic tools. Incorporating a theoretical framework in extension research allows us to understand the cognitive factors (expectations, efforts and social influences) that affect the adoption process, allowing these to be incorporated into an extension program [12].

This systematic review aims to explore, on a global scale, the methodology and results of extension research that seeks to promote genetic improvement in cattle. The research questions that drive this review are: (1) What are the most used research and extension methodologies to determine the factors that contribute to the adoption by farmers of practices and tools to promote genetic improvement in cattle? (2) What are the factors with the most effect on the adoption of genetic tools? (3) What is the practical impact of the extension activities?

## 2. Materials and Methods

The methodology applied in this analysis was adapted from the Preferred Reporting Items for Systematic Reviews and Meta-Analyses, PRISMA [22], see Supplementary Material.

### 2.1. Search Strategy

Due to the wide scope of terminology used when referring to extension activities and programs and their target audience, the search terms for this review aimed to capture the largest number of documents. The terms reflect the extension approach, the area of study, the target audience and the production sector, with special emphasis on the beef industry but without neglecting other cattle enterprises. The search terms were (extension OR education OR learning OR perception OR adoption OR “decision making” OR management OR attitude* OR behavior OR acceptance) AND (genetic* OR phenot*) AND (farmer* OR producer* OR grazier OR rancher OR adviser OR consultant) AND (beef OR livestock OR cattle). In addition, the use of * in terms allows to broaden the search results.

Search terms were used in four databases: title, abstract and keyword (TITLE-ABS-KEY) parts of documents in the Scopus database, and in the abstract (ab) on EBSCOHost, ProQuest and Web of Science on 4 July 2023. EndNote^TM^ 20 software was used to analyze the results found in the databases. As a result of the searches, 460, 34, 596 and 271 results were shown for Scopus, EBSCOHost, ProQuest and Web of Science, respectively.

### 2.2. Document Selection

**Figure 1:**
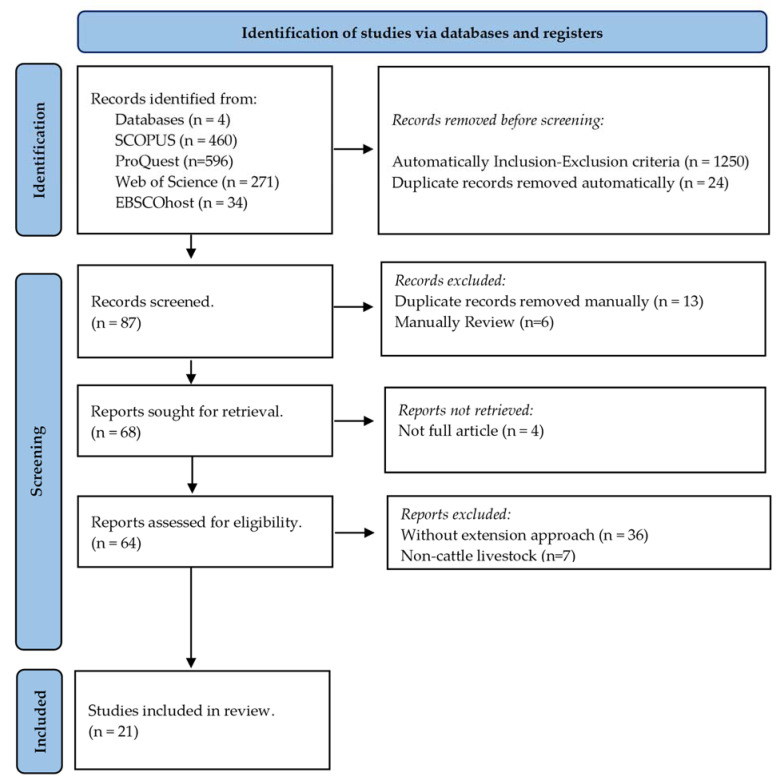
The search protocol and the resulting inclusions and exclusions. Adapted from Page et al., 2021 [22].

To refine the search results, inclusion and exclusion criteria were applied at an abstract level (Table 1). Where possible, documents were removed using an EndnoteTM20 automated tool, removing a total of 24 duplicates and 1250 articles based on words contained in their title (Table 1). The articles were then manually reviewed using an Excel (Microsoft^®^ Excel^®^ for Microsoft 365 MSO (Version 2310 Build 16.0.16924.20054) 64-bit) spreadsheet file, resulting in a final 21 articles for analysis (Figure 1).

Information was then extracted from each paper that allows the research questions mentioned above to be answered. This was collated in a matrix in an Excel spreadsheet with the most relevant information summarized, including: aim, main topic, contribution to genetic improvement, research extension approach, sampling, methods, data analysis, target audience, results and conclusions.

## 3. Results and Discussion

### 3.1. Location, Farmer Profile and Cattle Livestock

The geographical distribution of the studies of the selected documents shows a dispersion around the world. Australia is the country with the highest frequency (*n* = 3), followed by Ethiopia and Burkina Faso (*n* = 2) and finally Belgium, Bolivia, Brazil, Denmark, Indonesia, Mali, Mexico, Nigeria, Peru, Rwanda, Senegal, Somalia, Sweden and the US (*n* = 1). Sixty-six percent of the studies were published after 2019, indicating a growing interest in this area of research. In the selected articles, the farmers’ profiles are predominantly smallholders and medium-scale production systems (*n* = 14; 66.6%). The smallholders are usually focused on supporting themselves through family labor and consuming at least part of the production internally [23]. Among the articles that consider small- or medium-scale producers as the object of study, they are focused on beef [24,25], dairy [18,26,27], dual-purpose [27,28,29] and general cattle [30,31,32,33,34,35] production systems. In contrast, large-scale production was considered in six articles (28.6%) and examined only beef [36] and dairy [37,38,39,40,41] systems.

**Figure 2:**
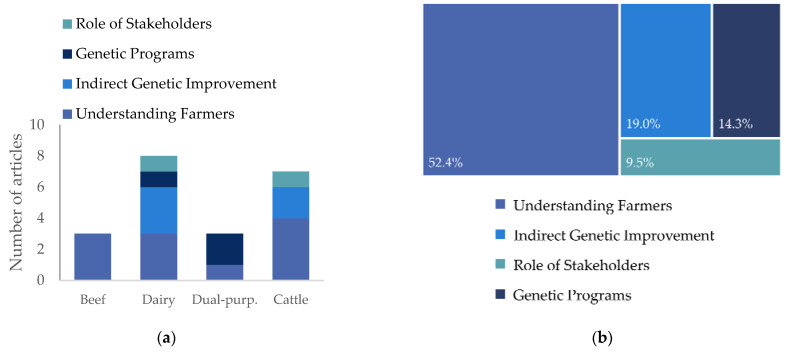
(**a**) Number of articles according to the cattle industry and its general scope, (**b**) proportion of articles classified according to their general scope with respect to genetic improvement in cattle.

The target species in this systematic review are cattle for beef production; however, the search terms included livestock and cattle to ensure all relevant articles were captured. Subsequently, livestock productions other than cattle (e.g., poultry, goat, pigs, sheep) were excluded from analysis. For those studies that considered cattle and other livestock [31,36], only the information and results on cattle were considered for this review. As shown in Figure 2a, 38.1% (*n* = 8) of the articles are related to understanding the use of genetic tools by dairy farmers [18,26,37,38,39,40,41,42]. Only 14.3% (*n* = 3) of the articles examine beef production [24,25,36], increasing to 61.9% when production with breeds for dual purpose (*n* = 3) were considered or when the authors did not make a distinction and referred just to cattle more generally (*n* = 7). The diversity of production systems and breeds involved (Angus, Hereford, Simmental, Asturiana de Valles, Avileña-Negra Iberica, Morucha, Parda de Montaña, Pirenaica, Retinta, Rubia Gallega, Creole, Holstein, Jersey, Lobi Taurine, Fulani Zebu, N’Dama Taurine, Sokoto Gudali, North Somali Zebu and crosses) can be explained by the diversity of geographic locations of the selected studies and the scale of production. The study of the adoption of genetic tools to aid selection is lower in beef compared with dairy cattle.

The selected articles were classified into four groups according to their general scope with respect to genetic improvement (Figure 2b). More than half of the articles are classified in the *Understanding Farmers* subgroup. These articles seek to understand the decision-making process by farmers or the factors that influence the adoption of tools/practices and enable genetic improvement. Articles whose main objective of the study was to analyze the adoption of practices or tools, such as artificial insemination (AI) [18,26,32,35] or sexed semen [37] when applied in production systems, which would lead to genetic improvement were classified in the *Indirect Genetic Improvement* subgroup. Three of the twenty-one articles were classified as *Genetic Programs*, which analyze the current situation [29,42] and provide insight [27] into the adequacy of specific breeding programs. The main objective of the remaining two articles [26,43] was to analyze the *Role of Stakeholders* and consultants, their impact on breeding programs in the cattle industry and the provision of technologies.

### 3.2. Methods and Frameworks

To answer the first research question from this review about what the most used research methodologies are to determine factors influencing the adoption of genetic tools, the information extracted from the selected studies is described below (Table 2 and Table 3). The articles that were classified within the *Understanding farmers* (*n* = 11) and *Indirect Genetic Improvement* (*n* = 4) groups are considered because their aims were related to understanding the process of adoption. Data collection was directly from the farmers, except for one study [33] that used data from a database of farmers who participated in an enhancement program.

Data collection from farmers was through surveys or interviews with the objective to capture information that allows the farmers and production systems to be characterized and to capture their attitudes and beliefs regarding the adoption of genetic and breeding tools. For instance, attitudes were collected through a series of statements that farmers could answer using a scale of agreement or disagreement [24,36,39,40]. Data collection from farmers was predominantly through surveys. However, interviews were used on three occasions in populations from Ethiopia [18], Indonesia [35] and Burkina Faso [24] to facilitate the completion of the questionnaire. The average number of farmers who responded to the questionnaire was 188 ± 152 responses across all studies. The sampling techniques found belong to the two major divisions of techniques in social sciences: probabilistic and non-probabilistic [44]. In the probabilistic sampling method, every farmer in the population of study has a chance (statistically known) of being selected, with a random selection procedure. In contrast, with the non-probabilistic technique, some farmers in the population have no possibility of being selected. This is because farmers are selected by non-random criteria (convenience or quota). This explains why non-probabilistic techniques may be subject to greater sampling bias relative to probabilistic techniques. The choice of one or another technique depends on the objective of the study. Probability sampling is beneficial when seeking an accurate description of the population, but non-probability sampling could be more useful for exploratory research.

#### 3.2.1. Attitudes toward Genetic and Breeding Tools

The studies analyzed farmers’ attitudes towards the use of different genetic and breeding tools through quantitative and qualitative methods, with mixed methods predominating in the research design. For the quantitative data, the studies conducted descriptive analyzes to present an aggregation of the surveyed data (e.g., response rate, data aggregated by region, constructed categorical variables, characteristics of respondents). In addition, inferential analyses were also used to test hypotheses related to the effect and magnitude of the factors involved in the attitude to the adoption of genetic tools. General linear models (GLM) are statistical procedures (e.g., regression models) widely used in the social sciences which allow conclusions to be reached about associations between variables [44]. In conjunction with qualitative analysis, it seeks to understand how variables affect the process of adoption through analysis of text (e.g., content analysis), data from interviews or surveys. Additionally, Likert scales are used to measure agreement or disagreement by farmers to statements related to the use of genetic tools or breeding technologies.

The theoretical models and frameworks used to study adoption processes, and the factors that drive them, provide order and structure in the analysis [10]. Incorporating a theoretical framework in extension research allows the cognitive factors (expectations, efforts and social influences) that affect the adoption process to be understood, allowing these to be incorporated into an extension program [12]. However, only two of the articles included for this review are based on a conceptual model of adoption (Table 3). This includes Ooi et al. [38], who utilized the Theory of Planned Behavior (TPB) with dairy farmers. The TPB [45] is a theoretical framework which places special emphasis on attitudes, intentions and perceived significance. Ooi et al. [38] found that farmers had a wide range of opinions and attitudes about fertility and genetics in the process of sire selection and highlights that considering farmers’ beliefs could facilitate the design of successful extension activities that have the objective of increasing the use of EBVs. The other article is from Lund et al. [41], who used Diffusion Theory [46] to examine the general acceptance of the genomic and reproductive technologies by dairy farmers. The key aspects of this theory are to consider the adoption rate (the relative speed at which the innovation is adopted by end users) and identify the variables that determine it (e.g., perceived attributes of innovations; type of innovation decision; communication channels, nature of social systems and extent of change agent’s promotion efforts). The author concluded that a greater number of interactions with breeding-related consultants encouraged general acceptance of the technology and a greater likelihood of using it. The scarce use of a theoretical framework in the selected works is in line with what was reported by Montes de Oca Munguia et al. [10] “…the majority of adoption studies in agriculture include measures of attitudes without adhering to one particular theory” (p. 11).

The articles selected for this review consider a single or combination of different tools (Table 2). Farmers’ attitudes and adoption factors of artificial insemination (*n* = 7) as a reproductive tool that facilitates genetic improvement is the most common tool, followed by crossbreeding (*n* = 5) and the use of EBV/Selection Index (*n* = 4).

#### 3.2.2. Preferences of Genetic Traits

Quantitative methodologies were used to analyze the preferences of the genetic traits (Table 3). Data collection through direct interviews was the predominate methodology used. Most of the articles analyzed smallholder farmers’ preferences for traits in cattle in Somalia [34], Guinea [30], Burkina Faso [31] and Mali [25], while trait preferences in the dairy sector in Australia [40] were through surveys. Knowing the trait selection preferences for beef farmers is crucial for the design of strategies to encourage participation in breeding programs. Extension and knowledge transfer services would raise awareness among farmers about the importance of traits to choose in the genetic selection of animals for commercial use but would also increase the willingness to use genetic tools and management strategies. However, preferences for traits and productive objectives are heterogeneous among farmers and vary over time.

### 3.3. Adoption Factors of Genetic Tools

To answer the second research question of this review: What are the factors with the most effect on the adoption of genetic tools? the factors measured in the articles that were classified as *Understanding Farmers* were analyzed. These studies present a social approach, where the characteristics, attitudes and preferences of the farmers are related to the adoption process in relation to the use of tools or management practices that enable one to increase the genetic potential of cattle. This is in accordance with Montes de Oca Munguia and Llewellyn [47], who reported that this tendency to focus on the characteristics of those who make the decisions (adopters) would respond to the influence of theoretical frameworks with a behavioral approach within research. Table 4 shows the main measurements considered as adoption factors of the technologies or practices analyzed in the articles.

Farmers’ attitudes are a crucial factor in technology adoption as they determine usage intentions and inflect human behavior during the decision-making process. Attitudes are measured through the degree of agreement or disagreement with respect to a pre-established statement and these are directly related to the research topic. The topics are use of fertility estimated breeding values (EBV) [38], breeding strategies and farmer participation in extension services [24], breeding tools such as traditional, genetic and genomic selection [36] and genomic selection combined whit reproductive technologies [41]. Subsequently, the results obtained on attitudes are related to the profile of the farmers. This would generate a flow of information that could be used in the design and evaluation of extension activities. Martin-Collado et al. [36] highlight the need to standardize the measurement of farmers’ attitudes in order to carry out studies across time and to be able to make comparisons between groups of farmers, making it easier to identify the main factors for the adoption of selection tools. This is consistent with the results obtained by Zoma-Traoré et al. [24] and Ooi et al. [38], who concluded that the interaction between extension agencies and farmers is crucial to improve farmers’ participation in breeding programs and encourage the use of genetic tools.

Farmers’ preferences regarding breeds, traits and breeding tools affect decisions in animal selection processes and technology adoption. Among the selected studies, the preferences of farmers regarding animal traits are generally analyzed in production systems where smallholders make decisions in developing countries [25,30,31,34]. Under these circumstances, adaptive traits are preferred by farmers, with the location of production systems having an influence. The incorporation of new breeds [31] and crosses [25] to genetically increase productive traits is of particular importance because the genetic base is expanded and its variability is increased. In addition, Roessler et al. [31] discussed that the importation of breeds also results in the incorporation of breeding technologies such as artificial insemination (AI) and the need for training. However, Martin-Collado et al. [40] concluded that in dairy systems, preferences for cow traits are intrinsic to farmers beyond production systems. Similar to what was claimed in the section of this review that considers articles that examine farmers’ attitudes toward genetic and breeding tools (Section 3.2.1), relating preferences (both for animal traits and breeding tools) to the farmer’s profile would make it possible to design and carry out genetic improvement programs.

Even though the farmer’s demographic profile is considered in most of the selected studies as a variable that helps explain their attitudes or preferences, only two of all the selected studies relate the socioeconomic profile of farmers to the level of adoption of technologies, without considering behavioral aspects. Vasquez et al. [28] found that the variables that predicted the adoption of technologies for genetic improvement were: the level of knowledge of genetic tools, the genetic characteristics of the animals and, from an economic approach, access to financing and credit. In the same vein, González et al. [33] found that the level of economic income is an important factor in the technology adoption process.

As mentioned above, in the studies selected for this review, different regression models are used to evidence the effect and magnitude of the factors. The outcomes of articles with regression analysis are shown in Table 5. Among the statistically significant factors, aspects related to extension and training services, as well as economic conditions or access to finance, were of crucial importance (Table 5). However, there are discrepancies throughout the articles analyzed. For example, literacy appears to have had a significant effect on the AI adoption process in Ethiopia [18], but education does not appear as a significant effect (N/S) for the same reproductive tool in Indonesia [35] or the adoption of improvement technologies in Peru [28]. Associated factors such as age, sex or land tenure do not emerge as significant (S) factors. In more specific studies on the use of reproductive tools [37] and genomics [41], the current use of technologies in production systems is a factor that would explain the probability of incorporating new technologies (Table 5). The heterogeneity in the factors that determine the adoption of tools that allow the genetic improvement of herds would be widely influenced by both the production systems and sociocultural conditions specific to each location. Therefore, it would seem more reasonable to make longitudinal comparisons in the same reference site to analyze temporal relationships between variables that explain the adoption processes and not between different locations in similar periods. Before designing extension activities, it may be crucial to have a deep understanding of the factors that affect farmers’ decision making in a specific productive area or region.

### 3.4. Impact on Genetic Improvements Programs

**Figure 3:**
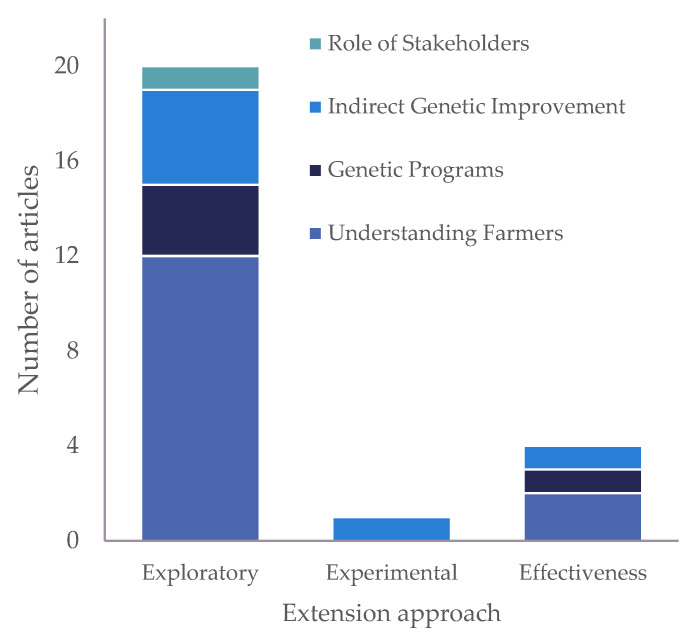
Number of articles according to the extension approach by general scope.

As agricultural extension and strategies policies have been driven by literature that usually does not offer practical advice on the variables that can be used to design interventions regarding adoption processes [47], this point is particularly relevant. In response to the third research question, “What is the practical impact of these extension activities?”, most of the published material focused on genetic improvement with an extension approach as the preliminary exploratory research method. This includes investigating the motivations, barriers and factors that affect the transfer of knowledge without an evaluation of extension activities (Figure 3). Nevertheless, several authors highlighted the importance of exploratory research results as a source of information for the development of extension activities [24,35,37,38]. Knowing the regional context in terms of genetic resources and knowledge through networks of stakeholders or agents of the production system seems to be crucial for the design of new extension programs that aim to promote genetic improvement.

Due to the exploratory nature of the articles selected for this review, with a particular focus on genetic tools in cattle, it is not possible to compare diverse extension methods. However, an extensive review on the effectiveness of extension methods in the global agricultural sector is addressed by Nettle et al. [15]. The author reviews result from different methods of extension such as facilitated groups/farmer-led groups/small-group learning; technology development, training, information provision, consultancy, e-extension, co-innovation, best management practice and social marketing. It is essential to obtain positive results in an extension project in terms of changes in decision-making processes, considering the points of view and challenges faced by farmers. Aligned with this, and highlighting the importance of having information available for future breeding strategies, Chagunda et al. [42] show that the majority of dairy farmers in the Girinka program (“One cow per poor family Program” in Rwanda) did not know the real breed of their cow. The author concludes that this lack of knowledge was an important barrier in possible genetic improvement programs.

Extension activities seem to be more efficient when they also incorporate other production issues that challenge farmers. For instance, Hatew et al. [26] suggested experimenting with different types of training for farmers with an integrated approach that goes beyond genetic improvement and considers aspects related to animal feeding. This is the only article selected for this review that experimented with different types of training. In another article, Camara et al. [43], through surveys of international experts in breeding programs, concluded that breeding programs need to consider the divergent points of view of stakeholders in their design and development. Therefore, knowing the regional context in terms of genetic resources and knowledge through networks of stakeholders or agents of the production chain is crucial for designing new extension programs that aim to promote genetic improvement. The effectiveness of breeding programs or extension activities has been considered in four articles of this review [24,35,37,38] However, there is no clear pattern due to the differences in objectives, including the effectiveness of types of training [26], participation in breeding programs [24,27], and productive programs [33]. This highlights the need for extension research beyond the exploratory approach, putting into practice and validating the results obtained through an extension program and subsequently analyzing its effectiveness.

## 4. Conclusions

The purpose of this systematic review was to explore on a global scale the methodology and results of extension research that seeks to promote genetic improvement in cattle. The studies used quantitative and qualitative methods with mixed methods predominating in the research’s design. Although social aspects were considered drivers in the adoption processes, the use of theories or frameworks from a social sciences perspective was incipient. The collection of information through surveys and interviews was widely used and accepted. The adoption of practices and technologies related to genetic improvement and preferences regarding traits to be selected in improvement programs vary according to the type of farmer and the production systems. Therefore, knowing the factors that encourage the adoption of technologies or processes is key in designing extension programs that seek to encourage genetic improvement through the application of traditional and new technologies. The participation of stakeholders and the cooperation of farmers in the design of extension services is crucial to know the topics in demand and the preferred strategies to encourage the use of genetic tools in cattle production systems. The data available from the articles selected for this review come from productively and culturally diverse circumstances, which limits generalized conclusions about strategies to motivate the use of genetic tools by beef farmers. Consequently, to carry out successful extension activities that seek to encourage the use of genetic tools in livestock farming for a specific productive region, it is imperative to conduct additional research with a participatory approach incorporating the viewpoints of commercial farmers and stakeholders.

## Figures and Tables

**Table 1:** Criteria used to maintain or discard documents in the systematic review.

Inclusion Criteria	Exclusion Criteria
Peer-reviewed journal articles with original empirical research. Articles published in English. Published between 2012 and the search for this review on 4 July 2023. The title of the article must contain one of the following words: extension, education, program, training, support, adoption, attitude, preference and acceptance.	Books, magazine articles, reports, newspaper articles, thesis, conference proceedings, generic, book sections, serial, case. Studies that do not consider practices that lead to genetic improvement in livestock systems. Studies that consider non-cattle livestock.

**Table 2:** Methods and analysis used in the selected articles regarding to attitudes toward genetic and breeding tools.

Tools	Framework	Sampling (Sample Size)	Data Collection	Data Analysis	Reference
AI	NA	No probabilistic (*n* = 71)	Survey: − Demographics and technical	− Descriptive statistics − Content analysis	[32]
		Multistage technique: purposively, random and systematic (*n* = 204)	Farmer interview Focus group	− Descriptive statistics − Tobit Model Regression − Narrative- Content analysis	[18]
		(*n* = 85)	Cross sectional interview	− Linear Multiple Regression	[35]
AI Natural mating Purebred Crossbreeding	NA	Purposively, random (*n* = 227)	Data from database	− Multivariate analysis − Cluster analysis	[33]
AI Purebred Crossbreeding Recording data Appearance Sharing bull Benchmarking	NA	Purposively, stratified (*n* = 125)	Survey/Interview: − Farming system, − Farmer profile, − Farmer breeding strategies, − Breeding tools.	− Descriptive statistics − Six-level Likert scale − PCA	[24]
AI Purebred Crossbreeding Appearance Recording data EBV DNA/gene data Benchmarking Embryo transfer	NA	Through breed associations (*n* = 328)	Survey: − Attitudinal statements, − Farming system, − Farmer profile, − Farmer breeding strategies, − Breeding tools.	− Six-level Likert scale − Reliability—Cronbach’s α − PCA − Validity—the Kaiser–Meyer–Olkin (KMO)	[36]
EBV	Theory of Panned Behavior	Purposively (*n* = 35)	Survey: − Demographics, − Interview, − Attitudes, − Subjective norms, − Perceived barriers.	− Descriptive statistics − Template analysis	[38]
EBV Selection Index	NA	Partly randomly (*n* = 551)	Survey farmer and farm profile	− Five-level Likert scale − Cluster analysis − ANOVA	[40]
Sexed semen Beef semen Genomic testing Crossbreeding Embryo transfer	NA	(*n* = 204)	Survey − Demographics and general, − Discrete choice experiment, − Statements.	− Seven-point scale − Descriptive statistics − Conditional Logit Model	[39]
Sexed semen Beef semen	NA	Partly random (*n* = 141)	Cross sectional survey	− Single or multiple choice − Descriptive statistics − Lineal Regression − Logistic Regression	[37]
Genomic selection ovum pick-up In vitro production of embryos	Diffusion Theory	Purposively, stratified (*n* = 175)	Interview Survey	− Descriptive statistics − Logistic Regression	[41]
Selection tools	NA	Probabilistic and used stratified random sampling (*n* = 144)	Survey − Social − Economic	− Bivariate correlations − Logistic Regression	[28]

PCA: Principal Component Analysis, NA: Not Applicable.

**Table 3:** Methods and analysis used in the selected articles regarding to preferences of traits.

Traits	Sampling (Sample Size)	Data Collection	Data Analysis	Reference
Body size Coat color Conformation Crossbreeding Disease resistance Docility Fast growing calves Fertility Milk yield Traction ability	Purposively, representative of two farmer group (*n* = 160)	Focus group Interview	Descriptive statistics Exploded Logit Model	[25]
Adaptation Fat Milk production Meat production Milk production Reproductive performance	Purposively, random (*n* = 11)	Participatory rural appraisals	Descriptive statistics	[34]
Appearance Behavior Body size/growth Breed Coat color/pattern Dam’s milk yield Pedigree	Purposively, snowball (*n* = 49)	Interview	Descriptive statistics Analyses of variance	[31]
Buying bulls Calving difficulty Cow live weight Feed efficiency Fertility, longevity Lactation persistency Lameness Mammary system Mastitis resistance Milking speed Protein yield Temperament	Partly randomly (*n* = 551)	Survey: − Farmer’s preferences, − Farmer and farm profile.	Descriptive statistics Pairwise comparison PCA and Cluster analysis Five-level Likert scale	[40]
Body conformation Body size Calving interval Disease resistance Heat tolerance Milk yield Survival	Purposively, random (*n* = 144)	Semi-structured interview	Chi-square (χ2) statistic Comparison	[30]

**Table 4:** Measurements considered in articles that analyze the adoption of technologies or practices that lead to genetic improvement in cattle.

	Technology/Practices	Measurements	Reference
Attitudes	Breeding tools	Attitudinal statements in farming systems, farmer profile, farmer breeding strategies and breeding tools.	[24]
		Attitudinal statements in traditional selection, genetic selection and economic selection.	[36]
	Reproductive technologies and genomic selection	General acceptance, likelihood of use, self-reported lack of understanding, perceived utility and ethical reservations.	[41]
	Fertility EBV	Attitudes, subjective norms and perceived barriers. Land tenure, age, gender, input of concentrate feeding, breed, AI sire selection method, characteristic of herd management and breeding objectives.	[38]
Preferences	Traits and Breeds	Household characteristics, herd composition, breeds, reasons for changing breeds and intended breed choice for the next 5 to 10 years.	[25]
		Gender, productive objectives, perception on traits, criteria to selecting male breeding, age of selection and culling of female animals.	[34]
		Age, household size, herd/flock size, gender, education level, ethnicity, main occupation, farm location, motivation to rising cattle or sheep, general breeding management, breed and traits preferences, selection criteria, culling decision and reasons.	[31]
	Traits	Farmer and farm profile, farmer attitudes toward breeding tools and criteria to selecting bulls.	[40]
	Traits and Breeding practices	Sex, marital status, education, primary occupation, access to credit, personal saving, type of landholding, age, household size, family members, size of land, experience, knowledge of husbandry practices, ranking of production objectives and reason for keeping cattle.	[30]
	Breeding tools	Geographical location, production systems, number of cows, production level, breeding management, gender, age, education, role on the farm, breeding interest and choice of breeding tools.	[39]
Farmer Profile	Selection tools	Characteristics of the producer, migration, characteristics of the herd, production system, level of organization, access to information and level of knowledge, economic characteristics of the producer, access to sources of financing and access to the market.	[28]
	Breeding practices and other technologies	Income, adopted technologies, schooling, vegetation coverage, feeding indicators, animal health, genetics and management.	[33]

**Table 5:** Summary of regression analysis results from articles selected for this systematic review.

Dependant Variables		Independent Variables	Effect
Tobit regression—Gebre et al. [18]			Marginal effect
AI rate	S	Literacy	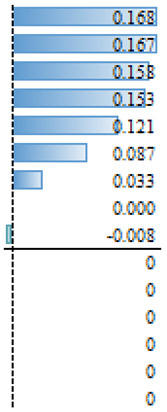
		Training	
		Feed supplementation practice	
		Access of the household to extension services	
		Mobile ownership	
		Number of cross breed cattle	
		Milk Yield	
		Income	
		Distance training center	
	N/S	Distance to AI service station	
		Gender	
		Age	
		Family size	
		Total farm size owned by the household	
		Total livestock holding in TLU	
Linear Multiple regression—Sirajuddin et al. [35]			β
Willingness to pay AI program	S	Social awareness	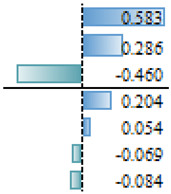
		Extension (counseling)	
		Location	
	N/S	Knowledge about AI	
		Education	
		Age	
		Business scale (herad)	
Logistic regression—Vazques et al. [28]			Odd Ratio
Adoption of technologies for genetic improvement	S	Genetic improvement tools	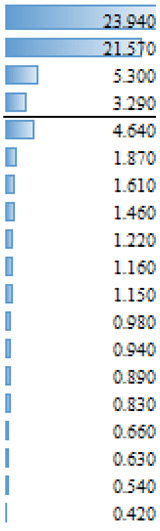
		Acces to finance	
		Genetic characteristics of animals	
		Credit Companies	
	N/S	Organizational level	
		Heads number	
		Educational level	
		Recognizes animal enhanced	
		Feeding	
		Livestock marketing	
		Livestock breeds	
		Milk production	
		Technical assistance	
		Technical assistance II	
		Area of land available for Livestock	
		Land tenure	
		Principal economy activity	
		Knowledge	
		Herd decision	
Logistic regression—Pereira et al. [37]			Odd Ratio
Use of beef semen in dairy	S	Sexed dairy semen use	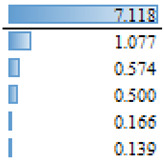
	N/S	Region 2	
		Herd breed	
		Herd size (>1500)	
		Herd size (501–1500)	
		Region 1	
Logistic regression—Pereira et al. [37]			Odd Ratio
Use of sexed dairy semen	S	Herd size (>1500)	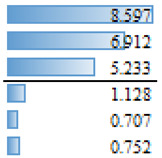
		Beef semen use	
		Herd size (501–1500)	
	N/S	Region 1	
		Herd breed	
		Region 2	
Logit model (likelihood)—Lund et al. [41]			Coefficient
	S	Perceived utility	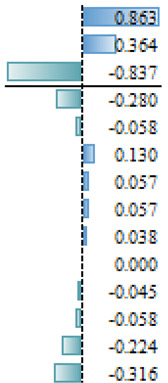
Use of the ovum pick-up–in vitro production of embryos–genomic selection technology		Frequency of use of NTM semen	
		Self-reported lack of understanding of the technology	
	N/S	Uses AI5	
		Number of communications with breeding consultant	
		Age 40–59 yr (ref 20–39 yr)	
		Proportion of purebred cows genomically tested	
		Keep up with new breeding technologies	
		Uses sexed semen	
		Farm size (number of cows)	
		Oganic Farm (idealistic reasin)	
		Organic Farm	
		Age 60 yr or more (ref 20–39 yr)	
		Ethical reservations	

S: Significant, NS: Not Significant.

## Data Availability

The data presented in this review are available within this article.

## Supplementary Materials

The following supporting information can be downloaded at: https://www.mdpi.com/article/10.3390/ani14020231/s1.
